# Surgical technique for the treatment of renal cell carcinoma with inferior vena cava tumor thrombus: tips, tricks and oncological results

**DOI:** 10.1186/s40064-016-1825-1

**Published:** 2016-02-20

**Authors:** Vital Hevia, Gaetano Ciancio, Victoria Gómez, Sara Álvarez, Víctor Díez-Nicolás, Francisco Javier Burgos

**Affiliations:** Urology Department, Renal Surgery and Kidney Transplant Section, Hospital Universitario Ramón y Cajal, Ctra Colmenar km 9,100, 28034 Madrid, Spain; Department of Surgery, Division of Transplantation, University of Miami Miller School of Medicine, Miami, FL USA

**Keywords:** Renal cell carcinoma, Inferior vena cava, Tumor thrombus, Surgical management

## Abstract

Renal cell carcinoma represents 3 % of all cancers. Around 4–10 % of cases present with inferior vena cava involvement, generally with tumor thrombus. Clinical and preoperative stage will be classified depending of the thrombus extension. A high quality preoperative workup is essential to properly plan surgical approach. Complete surgical resection of the tumor is potentially the only curative treatment, although it supposes a real challenge due to operative difficulty, potential for massive bleeding or tumor pulmonary thromboembolism. Surgery includes techniques derived from transplantation surgery and, in some cases, cardiovascular intervention with cardiopulmonary bypass. Long-term oncological outcomes after complete removal of the entire tumor burden are acceptable. In this report we describe step-by-step surgical maneuvers depending on the thrombus lever, and focusing in complete abdominal approach for the complete excision of the tumor. Moreover, a recent literature review about oncological results is reported.

## Background

Renal cell carcinoma (RCC) represents around 3 % of all cancers, with an incidence of 84,400 new cases per year in Europe (Ljungberg et al. 2015). It has a unique feature: its propensity for vascular invasion, extending through the renal vein and inferior vena cava (IVC). It occurs around 4–10 % of the cases, and in these cases complete surgical resection offers the only potential curative treatment. There has been a long debate around prognostic value of the thrombus extension, but a recent international multicenter study with more than 1000 patients has detected that the thrombus level is an independent predictor of survival (Martínez-Salamanca et al. 2014). Survival rates after radical nephrectomy and IVC thrombectomy also depend on the level of the thrombus, with a 5-year disease free survival of 64 % including all the levels (Ciancio et al. 2010). With such reasonable long-term survival, surgery is set as the only alternative for curative treatment, despite its high difficulty and its potential mortality (specially due to massive bleeding or pulmonary tumor thromboembolism).

Surgical approach varies among surgeons and thrombus level, although consensus is that these cases are complex, require an excellent knowledge of abdominal anatomy and benefit from a surgical team approach. In this report, we describe in detail the surgical technique for the complete resection of RCC with IVC involvement.

## Preoperative workup

**Fig. 1:**
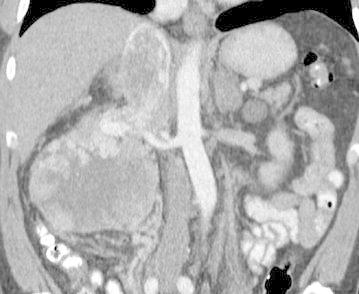
CT scan showing a large right renal cancer with IVC thrombus above hepatic veins but below diaphragm (IIIc)

**Fig. 2:**
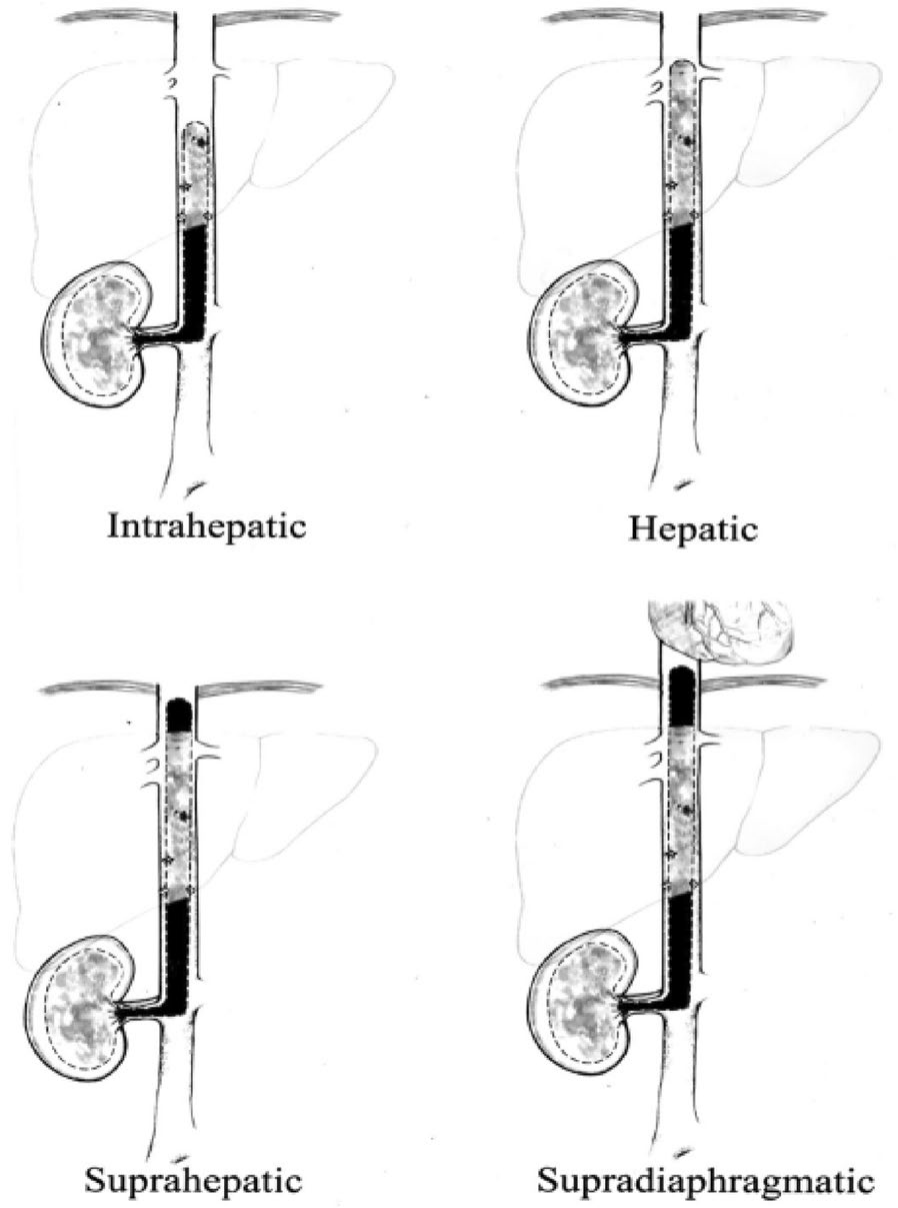
Subclassification of level III thrombus. IIIa: below hepatic veins. IIIb: reaching hepatic veins. IIIc: above hepatic veins. IIId: above diaphragm (from Ciancio et al. 2002)

The most crucial element in preoperative workup is to determine the level of the tumor thrombus (Fig. 1). In addition to its prognostic value, it will be essential for planning surgery. Most common tests used are computed tomography (CT) and magnetic resonance imaging (MRI). Both are useful for a correct staging, although venous involvement seems to be poorly defined on CT (Ljungberg et al. 2015). Neves-Zincke is the most common classification system to define the level of the thrombus (Neves and Zincke 1987). We routinely use a little modification for those thrombus level III, which are subdivided in 4 groups (IIIa, IIIb, IIIc and IIId) (Ciancio et al. 2002). To our knowledge, exposure and control of hepatic veins are a key factor in surgical strategy, so level III should be further subdivided depending on this needing. Thus, our classification according to thrombus extension is (Fig. 2):

- **I**: renal vein
- **II**: infrahepatic IVC
- **III**: retrohepatic IVC
  - *IIIa*: retrohepatic IVC below major hepatic veins
  - *IIIb*: retrohepatic IVC reaching the ostia of major hepatic veins
  - *IIIc*: retrohepatic IVC and extending above major hepatic veins, but below diaphragm
  - *IIId*: suprahepatic and supradiaphragmatic IVC, reaching intrapericardial IVC, but infra-atrial (outside right heart)
- **IV**: right atrium

Transesophageal echocardiography (TEE) is widely recommended for a correct staging of the level of the thrombus, especially for cases level III or IV. Although its use is sometimes avoidable in the preoperative workup, it will play an essential role during surgery, allowing the anesthetist and surgeon to have an intraoperative control of the thrombus, overall during clamping and thrombectomy steps. Moreover, it can help to early diagnose a potential tumor thromboembolism.

## Surgical technique

**Fig. 3:**
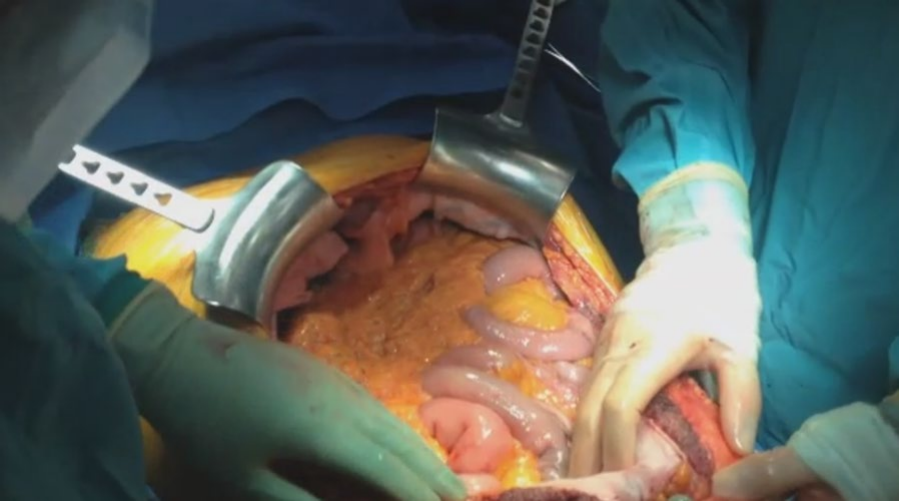
Chevron incision with Rochard retractor, enabling correct exposure of upper abdominal quadrants

Patient is placed in supine with extension of the lumbar lordosis in order to get a correct access to both subdiaphragmatic spaces. To gain optimal surgical exposure, we perform a bilateral subcostal incision (chevron) 3–4 cm below the costal margin, which sometimes it is extended in the midline vertically up to xiphoid process (triradiate incision). Rochard retractor is placed (Fig. 3), elevating costal margins and splaying them laterally. With this exposure liver mobilization or en bloc mobilization of the left upper quadrant abdominal structures (stomach, pancreas and spleen) will be easier.

### Early ligation of renal artery

**Fig. 4:**
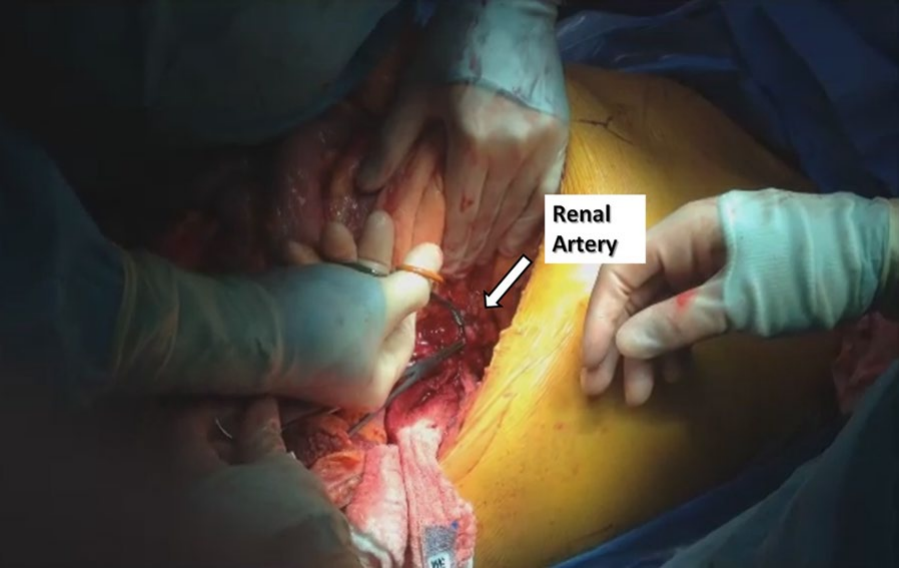
Early access and ligation of the renal artery during a left radical nephrectomy and IVC thrombectomy. The artery is dissected outside Zuckerkandl’s fascia and prior to perform the kidney’s anterior surface dissection

Kidney mobilization begins laterally and posteriorly, after opening parietal peritoneum, and mobilizing medially the kidney tumor until reaching the renal artery outside Zuckerkandl fascia. Once there, artery is ligated and divided (Fig. 4). The main advantage of this approach is that it encounters fewer collateral vessels than the classic anterior approach, and it allows an early collapse of collaterals circulation, reducing the bleeding and facilitating further dissection (Ciancio et al. 2003). To our knowledge, it is a good alternative to preoperative renal artery embolization and technically feasible.

### Level I tumor thrombus

Thrombus inside renal vein or reaching ostium in IVC can be resected safely with minimal dissection of IVC. After early ligation of renal artery, careful dissection of IVC 2 cm above and below the renal vein should be done. At this point, with a milking maneuver, thrombus can be pulled back to the renal vein and then a clamp is placed in the ostium. If circumstances do not allow bringing back the tumor into the renal vein, cavotomy and thrombectomy will be necessary. After clamping contralateral renal vein and the IVC above and below the thrombus, procedure can be done safely. It is important to note that every attempt to clamp the IVC should be preceded by a complete circumferential dissection and vascular control, in order to avoid massive blood loss. Abbasi reported that almost 40 % of cadaveric dissections presented a posterior lumbar vein (80 % men) draining in IVC above renal vein, contradicting classical anatomic texts assertion, and this unexpected vein was an unrecognized source of massive bleeding during surgery (Abbasi et al. 2012). Once the kidney is removed with the thrombus, closure of cavotomy is done with 4-0 Prolene running suture.

### Level II tumor thrombus

This level requires a more extensive vascular dissection, in order to get a correct exposure of infrahepatic and retrohepatic IVC. After early ligation of renal artery, liver’s posterior surface mobilization will be needed, exposing anterior and lateral surface of IVC. During this step, minor hepatic veins will be dissected, ligated and divided. Once again, total vascular control and circumferential IVC dissection are mandatory. Vascular clamps are placed in contralateral renal vein, below and above the thrombus, and then cavotomy and thrombectomy are performed safely. Nephrectomy is completed and closure of cavotomy is done with 4-0 Prolene running suture.

### Level III tumor thrombus

**Fig. 5:**
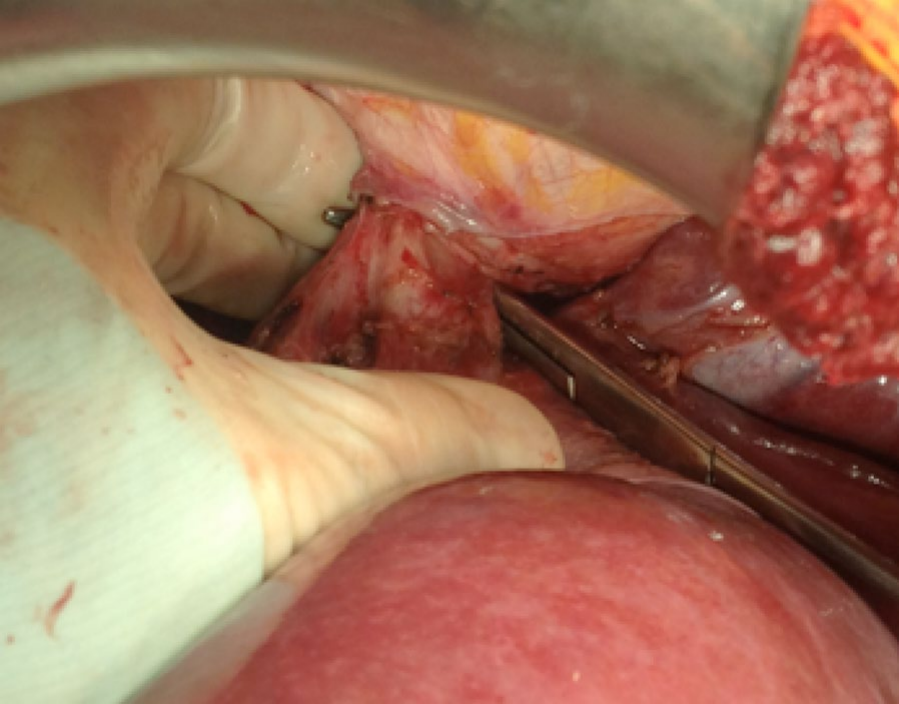
Piggy-back maneuver. Liver mobilization off the IVC, remaining attached by suprahepatic veins in their confluent with suprahepatic IVC

**Fig. 6:**
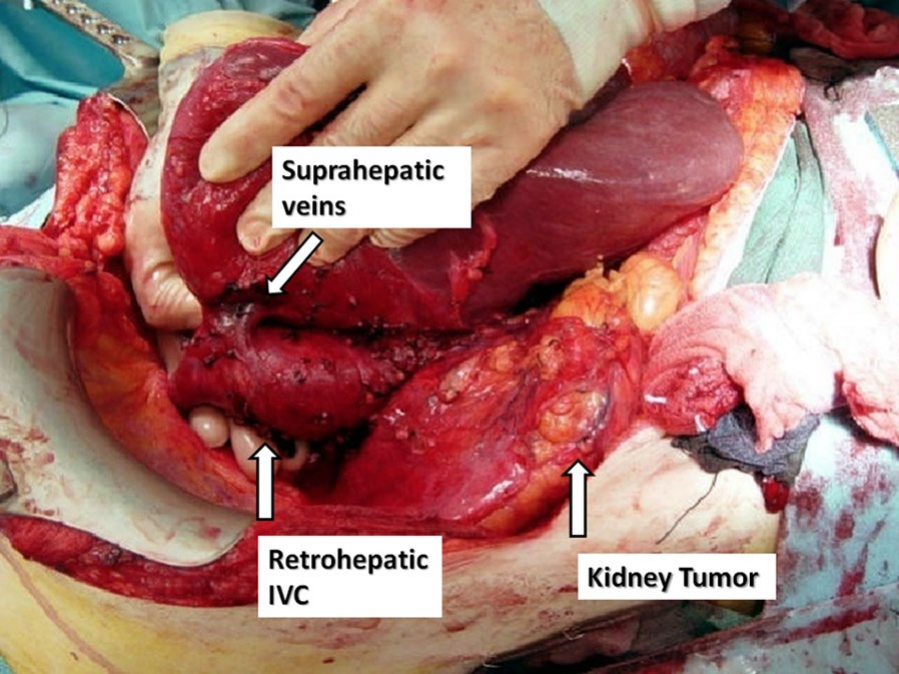
Complete liver mobilization and circumferential IVC dissection, gaining total vascular control. Minor hepatic veins draining from right and caudate hepatic lob are ligated

Given the close relation of the thrombus with the major hepatic veins and the retrohepatic IVC segment, a complete liver mobilization and IVC exposure is mandatory. Most of the surgical steps in this approach are derived from liver transplantation techniques. Mobilization begins with *ligamentun teres*, which is divided. After dividing falciform ligament, the incision is carried down to right superior coronary and triangular ligaments. Incision will continue with visceral peritoneum lateral to hepatic hilium, right inferior coronary ligament and hepatorenal ligaments. Thus, liver is softly rolled to the left abdomen, as previously described (Ciancio et al. 2011). Surgical control of the hepatic hilium is performed, allowing to use Pringle maneuver when needed (temporarily clamping portal vein and hepatic artery, mandatory if the thrombus reaches above hepatic veins). Then, “piggy-back” maneuver is performed (Fig. 5), as described for liver transplantation (Tzakis et al. 1989). It preserves IVC of the recipient, mobilizing the liver off the big vessel. To gain this total mobilization of the liver, minor hepatic veins draining on the IVC anterior surface are ligated and divided. In this fashion, the infrahepatic, retrohepatic and suprahepatic portions of the IVC are completely exposed, so the liver remains attached only by the hilium and the suprahepatic veins (Fig. 6). Finally, posterior surface of the IVC is completely dissected, in order to obtain the total circumferential dissection of the IVC.

As described for level I, but for some thrombus level IIIb/IIIc, a manual milking maneuver pulling the thrombus below the suprahepatic veins can be done, and the putting the clamp below them. This step should be assisted by TEE control, assessing about the level of the clamp and the potential dislodging of the thrombus and its subsequent pulmonary embolism. This milking maneuver has two main advantages: it avoids hepatic ischemia and it preserves liver drainage into the IVC through the suprahepatic veins (with an acceptable venous return). This technique is often feasible, especially when early ligation of renal artery was performed, because it reduces blood supply to the tumor thrombus. For level IIId thrombus, and for those cases when milking maneuver is not feasible, dissection continues until the supradiaphragmatic and intrapericardial IVC, which is dissected circumferentially.

Once with IVC dissected, thrombectomy is performed previous correct sequential vascular clamping: Pringle maneuver, IVC below the thrombus, contralateral renal vein, right adrenal vein if needed, and IVC above the thrombus (below suprahepatic veins if milking maneuver was successful or around supradiaphragmatic IVC if not). Cavotomy starts through affected renal vein and is extended proximally as much as needed. Tumor thrombus is removed completely, including adherent thrombus to IVC wall with sharp dissection. Major hepatic veins and IVC wall can be directly visualized; it allows complete removing of thrombus in their ostia. IVC closure is done with 2 Prolene 4-0 running sutures.

As previously commented, cases requiring hepatic ischemia and IVC clamping above hepatic veins have surgical disadvantages, due to intraoperative venous return decrease and to postoperative hepatic dysfunction. Thus, the less time the ischemia is established, the less risk of perioperative complications. That is the reason why we routinely recommend replacing clamps after thrombectomy. Once the thrombus is removed, a vascular clamp is positioned immediately above the proximal segment of the cavotomy, so the suprahepatic clamp and hepatic hilium clamp can be safely removed and then hepatic circulation reestablished.

### Level IV tumor thrombus

**Fig. 7:**
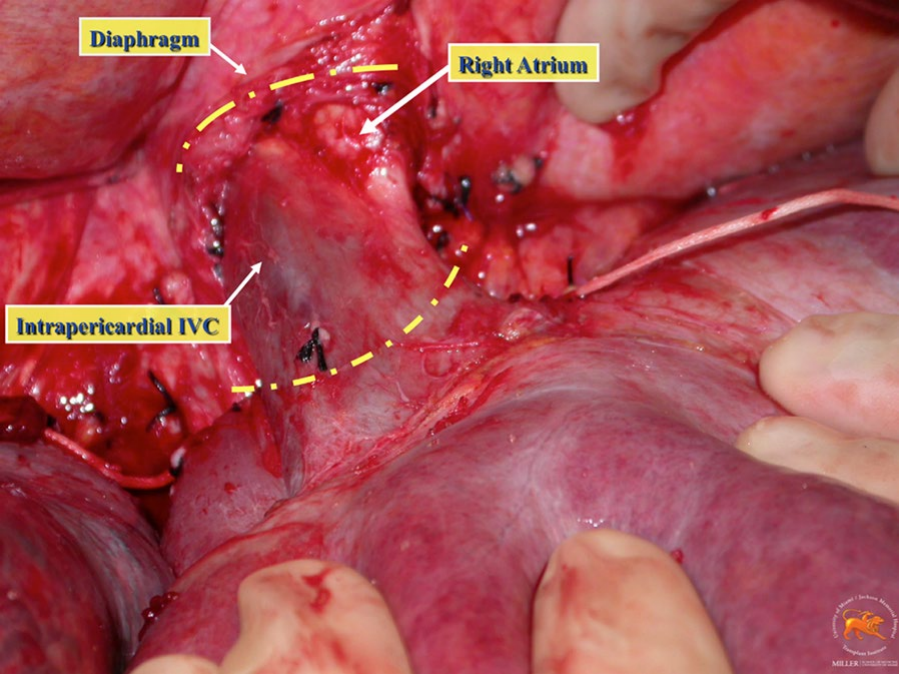
Supradiaphragmatic and intrapericardial IVC dissection, which will be necessary for level IIId and IV thrombus

**Fig. 8:**
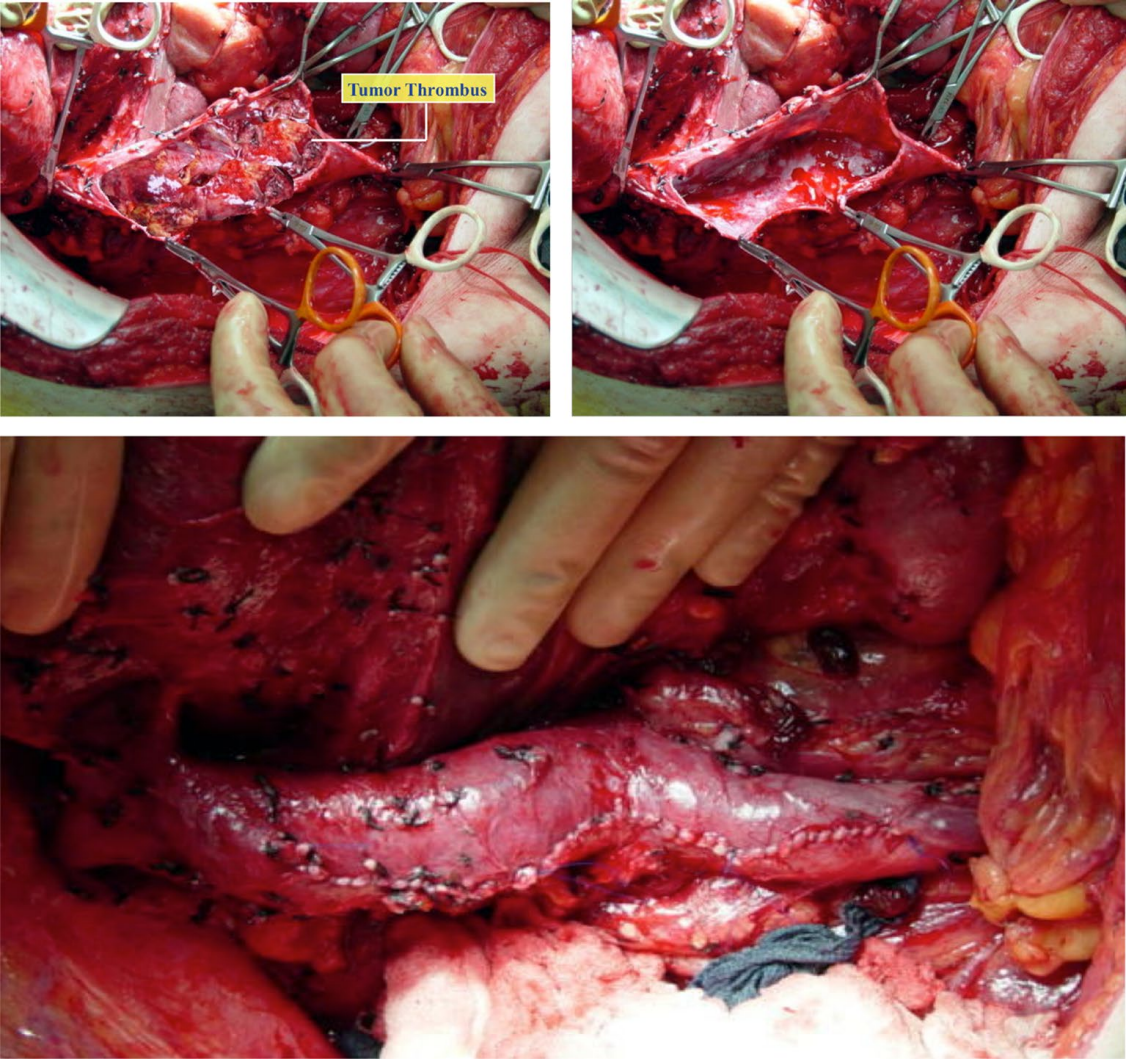
cavotomy and complete thrombectomy. Closure of the IVC is done with 4-0 non-absorbable running suture

Early surgical approach of level IV thrombus relied on cardiopulmonary bypass (CPB). It can produce platelet dysfunction and coagulopathy (Novick et al. 1990), with the consequent extensive bleeding, and it is not exempt of complications arising mainly from hypothermia. In our experience, CPB may be required in patients with a large and fixed atrial thrombus. In these cases, collaboration with cardiothoracic surgeons is necessary to remove all the thrombus from the right heart. However, patients presenting with a little and not adherent atrial thrombus may be resectable through a complete abdominal approach and would benefit from the use of liver transplantation techniques described previously. After opening the central tendon of the diaphragm, supradiaphragmatic and intrapericardial IVC is identified and dissected until it can be correctly encircled (Fig. 7). Once here, confluence of IVC in the right atrium can be gently pulled to the abdomen, bellow the diaphragm. Clamps positioning, cavotomy, thrombectomy and IVC reconstruction should be done in conventional fashion (Fig. 8).

### Need for IVC resection

Oncological success in RCC treatment will depend on the complete tumor removal. Sometimes there is no only tumor thrombus, but the IVC wall is infiltrated and has to be removed. It is quite difficult, but not impossible, to determine whether the IVC wall is invaded or not. The assessment of some radiographic features was associated with a significantly increased risk of IVC resection (Psutka et al. 2014). Although useful for a good preoperative planning, only surgical exploration has real value.

**Fig. 9:**
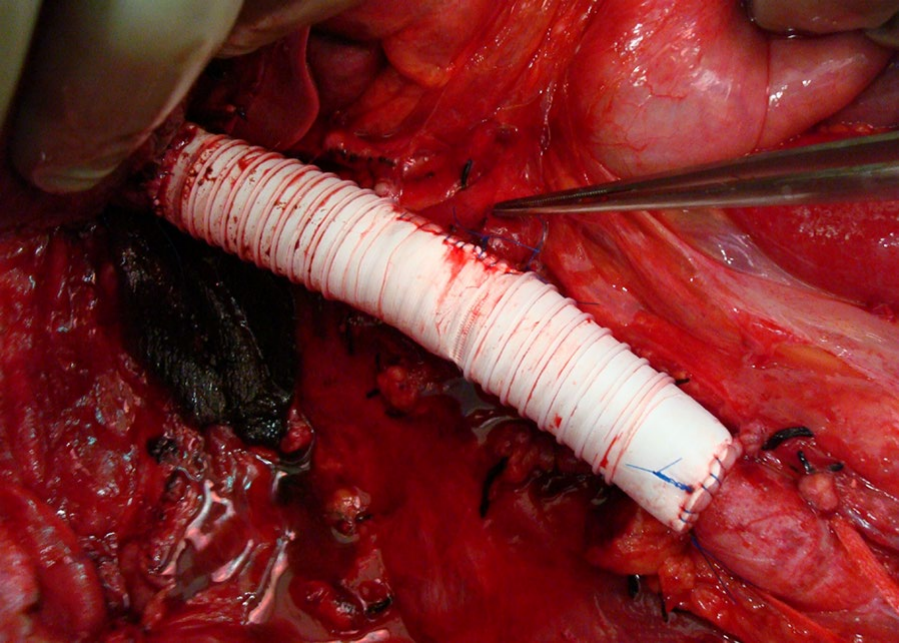
placement of a PTFE prosthesis after IVC resection, with end-to-side anastomosis to left renal vein

Resection of the IVC may not be necessary in most of the cases. Furthermore, complete “piggyback” liver mobilization facilitates complete thrombectomy of large thrombus, avoiding IVC resection. When IVC is affected by the tumor, the evaluation of collateral vessels is useful to decide what to do. If complete occlusion of IVC is present with chronic obstruction, an adequate blood flow through collaterals is presumed, creating a natural veno-venous bypass. En bloc resection of the IVC can be performed safely with low postoperative morbidity (Ciancio and Soloway 2005; Kearney et al. 1981). However, if collateral circulation is not present, resection of IVC should be avoided. In this setting, IVC reconstruction can be done with prosthesis, usually a PTFE graft (Fig. 9). Its main risk is thrombosis or infection (Caldarelli et al. 2002). Other options are autologous vein grafts or pericardium (Marshall and Reitz 1985).

## Oncological results

Complete surgical resection represents the mainstay of the treatment for RCC with IVC thrombus (RCCIVCT). Median survival for patients with RCCIVCT not undergoing treatment is 5 months (Reese et al. 2013). However, 5-year cancer specific survival for patients with RCCIVCT following radical surgery, in the absence of lymph nodes or distant metastases, is around 60 % (Ciancio et al. 2010; Kaag et al. 2011; Martínez-Salamanca et al. 2011; Kirkali and Van Poppel 2007). These results support surgical treatment in order to provide long-term cancer control, even for patients with locally advanced tumor thrombus and especially in the absence of distant metastases. At present, in the targeted therapies era, it is not yet well known the role that systemic therapies will play in the treatment of RCCIVCT. Several cases have been reported, such as downstaging a level IV with neoadjuvant Sunitinib (Karakiewicz et al. 2008). The real role of these new drugs should probably be investigated in the near future under randomized clinical trials.

Most controversial question regarding predicting factor of RCCIVCT is if the level of the thrombus is an independent factor of oncological outcomes. Several studies reported conflicting data. Recently, the largest multi-institutional and international cohort reported by Martinez-Salamanca (2011) with more than 1000 patients found that tumor thrombus level is an independent predictor of survival.

## Conclusions

Radical surgery is the mainstay of treatment for patients with RCCIVCT. And adequate preoperative imaging is essential. Surgical maneuvers and techniques derived from liver transplantation provide excellent exposure and control of the IVC, avoiding the use of CPB in many cases. Level of the thrombus seems to be a survival independent predictor. Successful surgery may provide acceptable long-term survival rates and better quality of life. New targeted therapies may play a promising role as adjuvant therapy.

## References

[CR1] Abbasi A, Johnson TV, Kleris R, Ying K, Bonner MY (2012). Posterior lumbar vein off the retrohepatic inferior vena cava: a novel anatomical variant with surgical implications. J Urol.

[CR2] Caldarelli G, Minervini A, Guerra M, Bonari G, Caldarelli C, Minervini R (2002). Prosthetic replacement of the inferior vena cava and the iliofemoral vein for urologically related malignancies. BJU Int.

[CR3] Ciancio G, Soloway M (2005). Resection of the abdominal inferior vena cava for complicated renal cell carcinoma with tumour thrombus. BJU Int.

[CR4] Ciancio G, Vaidya A, Savoie M, Soloway M (2002). Management of renal cell carcinoma with level III thrombus in the inferior vena cava. J Urol.

[CR5] Ciancio G, Vaidya A, Soloway M (2003). Early ligation of the renal artery using the posterior approach: a basic surgical concept reinforced during resection of large hypervascular renal cell carcinoma with or without inferior vena cava thrombus. BJU Int.

[CR6] Ciancio G, Manoharan M, Katkoori D, De Los Santos R, Soloway MS (2010). Long-term survival in patients undergoing radical nephrectomy and inferior vena cava thrombectomy: single-center experience. Eur Urol.

[CR7] Ciancio G, Manoharan M, Katkoori D, de los Santos D, Soloway M (2010). Long-term survival in patients undergoing radical nephrectomy and inferior vena cava thrombectomy: single-center experience. Eur Urol.

[CR8] Ciancio G, Gonzalez J, Shirodkar SP, Angulo JC, Soloway M (2011). Liver transplantation techniques for the surgical management of renal cell carcinoma with tumor thrombus in the inferior vena cava: step-by-step description. Eur Urol.

[CR9] Kaag MG, Toyen C, Russo P (2011). Radical nephrectomy with vena caval thrombectomy: a contemporary experience. BJU Int.

[CR10] Karakiewicz PI, Suardi N, Jeldres C, Audet P, Ghosn P, Patard JJ, Perrotte P (2008). Neoadjuvant sutent induction therapy may effectively down-stage renal cell carcinoma atrial thrombi. Eur Urol.

[CR11] Kearney GP, Waters WB, Klein LA, Richie JP, Gittes RF (1981). Results of inferior vena cava resection for renal cell carcinoma. J Urol.

[CR12] Kirkali Z, Van Poppel H (2007). A critical analysis of surgery for kidney cancer with vena cava invasion. Eur Urol.

[CR13] Ljungberg B, Bensalah K, Canfield S, Dabestani S, Hofmann F (2015). EAU guidelines on renal cell carcinoma: 2014 update. Eur Urol.

[CR14] Marshall FF, Reitz BA (1985). Supradiaphragmatic renal cell carcinoma tumor thrombus: indications for vena caval reconstruction with pericardium. J Urol.

[CR15] Martínez-Salamanca JI, Huang WC, Millan I (2011). Prognostic impact of the 2009 UICC/AJCC TNM staging system for renal cell carcinoma with venous extension. Eur Urol.

[CR16] Martínez-Salamanca JI, Huang WC, Millán I, Bertini R, Bianco FJ (2014). Lessons learned from the International Renal Cell Carcinoma Venous Thrombus Consortium (IRCC-VTC). Curr Urol Rep.

[CR17] Neves RJ, Zincke H (1987). Surgical treatment of renal cancer with vena cava extension. Br J Urol.

[CR18] Novick AC, Kaye MC, Cosgrove DM (1990). Experience with cardio- pulmonary bypass and deep hypothermic circulatory arrest in the management of retroperitoneal tumors with large vena caval thrombi. Ann Surg.

[CR19] Psutka SP, Boorjian SA, Thompson RH, Schmit GD, Schmitz JJ (2014). Clinical and radiographic predictors of the need for inferior vena cava resection during nephrectomy for patients with renal cell carcinoma and caval tumour thrombus. BJU Int.

[CR20] Reese AC, Whitson JM, Meng MV (2013). Natural history of untreated renal cell carcinoma with venous tumor thrombus. Urol Oncol.

[CR21] Tzakis A, Todo S, Starzl TE (1989). Orthotopic liver transplantation with preservation of the inferior vena cava. Ann Surg.

